# Genetic insight and mapping of the pod constriction trait in Virginia-type peanut

**DOI:** 10.1186/s12863-018-0674-z

**Published:** 2018-10-19

**Authors:** Abhinandan S. Patil, Sigal Popovsky, Yael Levy, Ye Chu, Josh Clevenger, Peggy Ozias-Akins, Ran Hovav

**Affiliations:** 1 Department of Field Crops, Institute of Plant Sciences, Agriculture research organization –the Volcani Center, HaMakkabbim Road, P. O. Box 15159, 7505101 Rishon LeZiyyon, Israel; 2 Department of Horticulture and Institute of Plant Breeding, Genetics and Genomics, The University of Georgia, Tifton, GA 31793 USA

**Keywords:** Virginia-type peanut, Pod constriction, Recombinant inbred lines, SNP-Array, Genetic mapping, Physical mapping

## Abstract

**Background:**

Pod constriction is an important descriptive and agronomic trait of peanut. For the in-shell Virginia marketing-type, this trait has commercial importance as well, since deeply constricted pods have a tendency to break, which makes them unmarketable. Classical genetic studies have indicated that pod constriction in peanut is controlled by one to four genes, depending on the genetic background. In all of those studies, pod constriction was evaluated visually as opposed to quantitatively. Here, we examined the genetic nature of this trait in the Virginia-type background. Our study involved 195 recombinant inbred lines (F_7_RILs) derived from two closely related cultivars that differ in their degree of pod constriction. Pod constriction was evaluated visually and quantitatively in terms of the pod constriction index (PCI), calculated as the average ratio between the pod’s waist and shoulders.

**Results:**

ANOVA and genetic parameters for PCI among the F_7_RILs in three blocks showed very significant genotypic effect (p(F) < 0.0001) and high heritability and genetic gain estimates (0.84 and 0.52, respectively). The mean PCI values of the different RILs had a bimodal distribution with an approximate 1:1 ratio between the two curves. Pod constriction was also determined visually (VPC) by grading the degree of each RIL as ‘deep’ or ‘slight’. The χ^2^ test was found to not be significantly different from a 1:1 ratio (*p* = 0.79) as well. SNP-array-based technology was used to map this trait in the RIL population. A major locus for the pod constriction trait was found on chromosome B7, between B07_120,287,958 and B07_120,699,791, and the best-linked SNP explained 32% of the total variation within that region. Some discrepancy was found between the SNPs original location and the genetic mapping of the trait.

**Conclusion:**

The trait distribution and mapping, together with data from F_1_ and F_2_ generations indicate that in this background the pod constriction is controlled by a major recessive gene. The identity of loci controlling the pod constriction trait will allow breeders to apply marker-assisted breeding approaches to shift allelic frequencies towards a slighter pod constriction and will facilitate future effort for map-based gene cloning.

**Electronic supplementary material:**

The online version of this article (10.1186/s12863-018-0674-z) contains supplementary material, which is available to authorized users.

## Background

Peanut (*Arachis hypogaea* L.) is known for the incongruity between its very narrow genetic polymorphism and the great phenotypic diversity among peanut cultivars [1, 2]. Domesticated peanut is allopolyploid (2n = 4× = 40) that is composed from two diploid species *Arachis duranensis* and *Arachis ipaensis*, named A and B genome. As in many polyploid species, cultivated peanut has experienced a genetic bottleneck which, superimposed with the effects of domestication and self-pollinating system, has greatly narrowed the genetic diversity and limited DNA polymorphism among subsequently derived *Arachis* forms [2]. The phenotypic diversity can be seen in peanut pods, which vary widely in their size, structure and texture. One of these highly variable traits, pod constriction, is an important descriptive trait used to distinguish between peanut market-types [3]. For example, Valencia (*fastigiata*) types are recognized by their very slight pod constriction; whereas Virginia (*hypogaea*) and Spanish (*vulgaris*) types usually have slight-to-moderate pod constriction. The *hirsuta* subspecies (*A. hypogaea ss. hirsuta*) characterized by deep to very deep pod constriction, particularly between the second and third seed segments. Pod constriction can also vary considerably within peanut types, especially within the Virginia-type group, in which the degree of pod constriction ranges from very deep to very slight.

Pod constriction is also an important biological and commercial trait. It influences seed development, since pods with very slight or no constriction usually bear tightly packed, flattened seeds that are in direct contact with their neighbors [4]. This may cause damage to the embryos and also increase the amount of seed-splitting during the shelling process. On the other hand, highly constricted pods are undesirable as well, especially for the in-shell industry, because they tend to carry dirt that detracts from the pod’s final tint. More importantly, they tend to split during the harvesting and sorting process, rendering the final product unmarketable.

Due to its commercial importance, the genetic nature of pod constriction has been the subject of several studies. In fact, pod constriction is one of the oldest genetically inspected traits, as it was one of the seven traits that Mendel [5] studied in pea. Mendel hypothesized that a single gene controls the pod constriction trait, in which the “inflated” pod phenotype is dominant over the “constricted” pod phenotype. Pod constriction is the only one of Mendel’s genetic traits that has not yet been cloned and this trait has received less research attention than any of the other traits he studied [6, 7]. In peanut, several classical studies were performed to address the inheritance of pod constriction and those studies led to the proposals of a two-gene model [8] and a three-gene model [9] in which slight constriction is dominant over deep constriction. A four-gene model (three unlinked nuclear loci and one cytoplasmic locus) was also proposed [10]. In that model, the deep constriction is dominant over slight constriction. A more recent study involving Spanish-type peanuts [11] reported that a single gene controls pod constriction, with deep constriction dominant over slight constriction. Also, the inheritance of pod constriction was analyzed by using a cross between a Spanish-type variety and its narrow-leaf mutant [12, 13], suggesting that pod constriction is under trigenic control (i.e., any two of the three complementary dominant genes lead to constricted pods). The identification of regions controlling the pod constriction trait will lead to fine mapping efforts to further the understanding of the genes controlling the trait.

In the present study, a recombinant inbred lines (RILs) population was analyzed to investigate the genetic nature of the pod constriction trait of peanut. The population is based on a cross between very closely related Virginia marketing-type cultivars that differ in their pod constriction. As mentioned above, pod constriction is an important trait in the Virginia-type in-shell industry. Yet, there is little information in the literature regarding pod constriction among Virginia-type germplasm. We evaluated pod constriction visually (VPC) and also quantitatively, by measuring the pod constriction index (PCI) in a random sample of pods. In addition, SNP-array-based technology [14, 15] was used to map the pod constriction, providing new insight into the genetic nature of this trait in peanut.

## Results

### Variance and genetic analyses of pod constriction among the RIL population

Variance and genetic parameters for PCI among 195 F_7_RILs in three blocks are presented in Table 1. As shown, the genotypic effect of the RILs on PCI was very significant (p(F) < 0.0001), while the effect of the block was non-significant (p(F) = 0.87). In the agreement, the heritability and genetic gain estimates were high (0.84 and 0.52, respectively). Accordingly, the further genetic and mapping analyses were done on the average PCI values.

**Table 1:** ANOVA and genetic parameters for PCI among 195 F_7_ RILs in three blocks

Source of variation	Degrees of freedom	Sum of Squares	Mean Squares	*F* ratio	Probability (*F*)
Block	2.00	0.00	0.00	0.19	0.87
RIL	194.00	24.29	0.13	19.31	< 0.0001
Error	388.00	2.52	0.006		
Genetic parameters					
ECV (%)		11.04			
GCV (%)		27.26			
PCV (%)		29.41			
h^2^ (Broad Sense) (%)		83.92			
Genetic Advancement (5%)		52.05			

ECV; GCV; PCV- environmental, genetic and phenotypic coefficient of variation, respectively. h^2^ - heritability

**Fig. 1:**
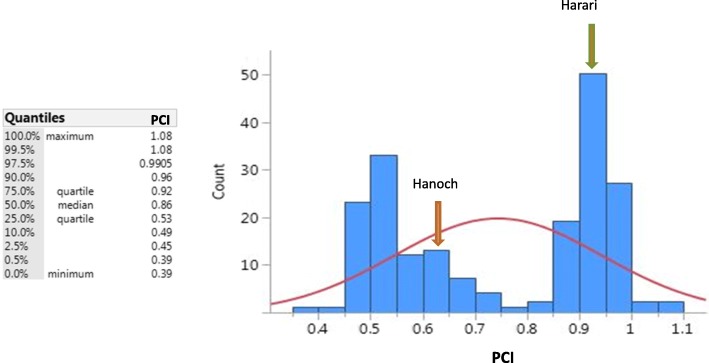
Distribution of the mean pod constriction index (PCI) trait in 195 RILs derived from the Hanoch X Harari cross. Count = number of RILs in this category

The distribution of the average PCI scores of the 195 RILs is presented in Fig. 1. Average PCI varied between 0.39 and 1.08. Yet, as shown, PCI was not distributed normally, but instead appeared to have a clearly bimodal distribution with some small overlap between the two curves. The median PCI was 0.86 with a small tendency toward the higher PCI values, suggesting an approximate 1:1 ratio between the two curves. The parental line values were located within each of the curves. Some deviation of the lower values from the value observed for cv. Hanoch was noted, illustrating an over-representation of the extra-deep pod constriction phenotype in the RIL population.

Pod constriction was also determined visually (VPC) by grading the degree of pod constriction of each RIL as deep (like ‘Hanoch’) or slight (like ‘Harari’). Out of the 195 RILs, 100 F_7_ RILs were rated as “deep” and 95 RILs were rated as “slight” (Additional file 1: Figure S1; Table 2). On the basis of the expected and observed frequencies of VPC, the χ^2^ test was found to not be significantly different from a 1:1 ratio (*p* = 0.79). VPC was also evaluated among Hanoch × Harari based F_1_ and F_2_ populations (a detailed description of those populations can be found in Kayam et al. [16]). VPC segregated at a rate of 5:0 for slight: deep among the F_1_ plants and at a rate of approximately 3:1 (*p* = 0.39) among the F_2_ generation (Table 2).

**Table 2:** Inheritance of pod constriction as determined visually (VPC) among the F_1_, F_2_ and F_7_ progeny of ‘Hanoch’ × ‘Harari’

Generation	Phenotype			Expected ratio	Observed ratio	χ 2 value	*p* value
	Total	Deep constriction	Slight constriction				
F_1_	5	–	5	–	–	–	–
F_2_	273	78	195	3:1	1:2.5*	0.74	0.39
F_7_	195	100	95	1:1	1.04:1*	0.07	0.79

*df* = 1. *The observed ratio is not significantly different from the expected ratio at *p* = 0.05

F_1_ and F_2_ plants were grown as described at [16]

**Fig. 2:**
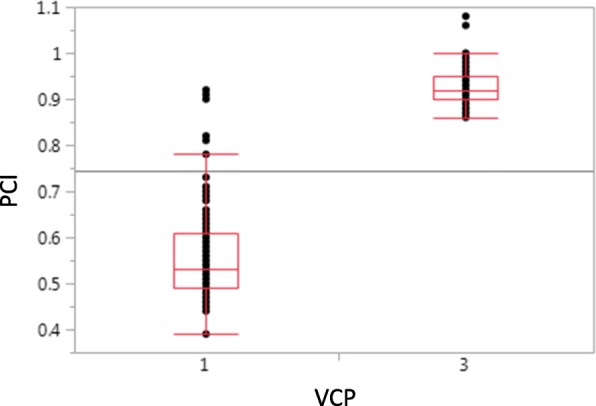
Comparison of quantitative and visual pod constriction of 195 F_7_ RILs. Quantitative pod constriction is the constriction index (PCI) based on quantitative measurements of the waist and shoulders of the peanut pod. Visual pod constriction (VPC) was rated on a scale of 1 (deep) to 3 (slight)

The association between VPC and PCI among the RILs was inspected (Fig. 2). In general, a strong relationship was observed between VPC and PCI, as reflected by the significant difference (p(t) < 0.0001; R^2^ = 0.84) between the average PCIs of the deep (0.56) and slight (0.93) VPC groups. Yet some points were outlined on the graph, especially from the deep VPC group, presenting high PCI values.

### Mapping of VPC and PCI

**Fig. 3:**
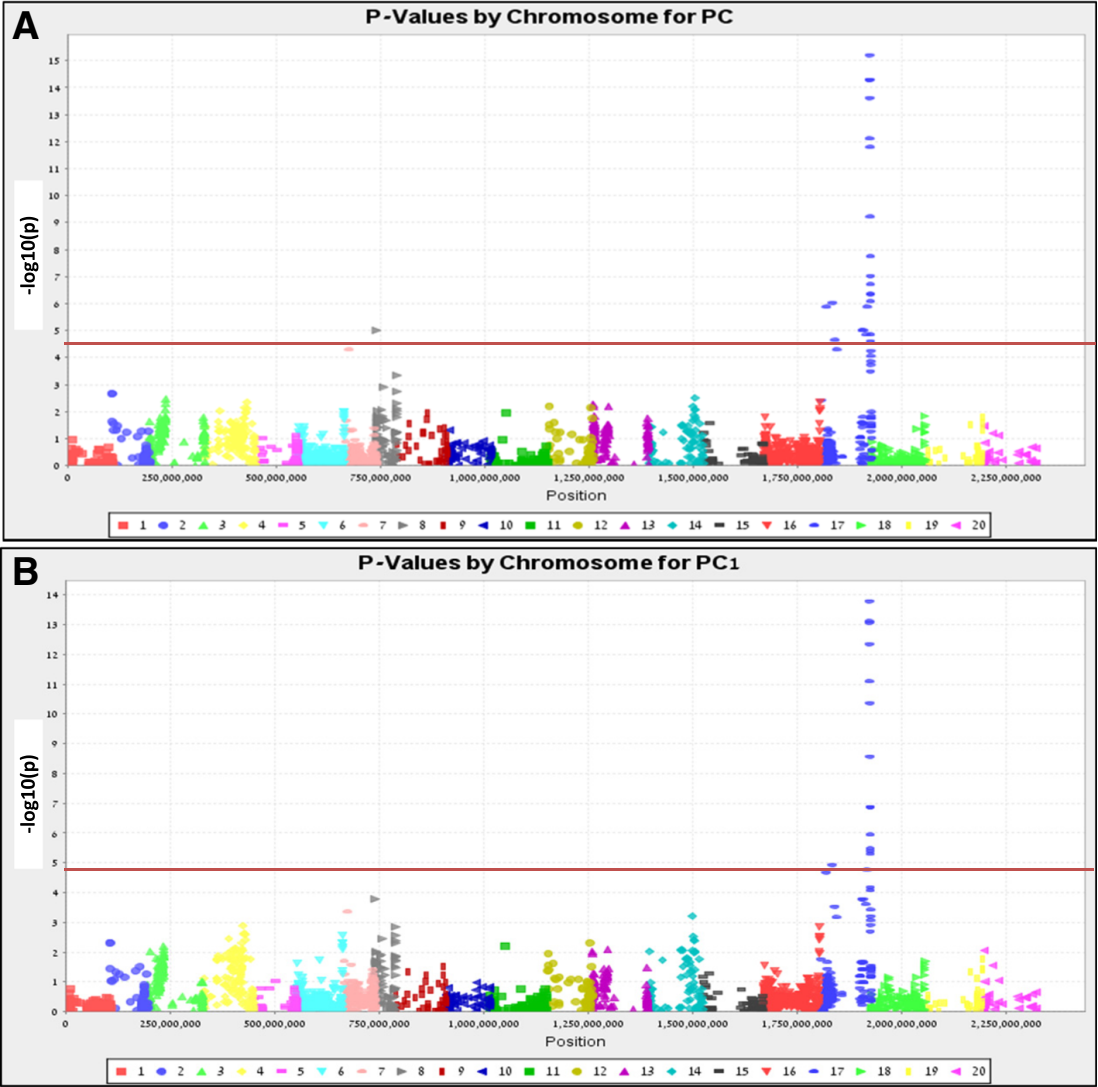
Microarray-based Manhattan plot showing a significant association between the SNPs for VPC (**a**) and PCI (**b**). The relative densities of SNPs physically mapped on 20 chromosomes of the peanut genome are plotted along the x-axis. 1–10 = genome A (1–10); 11–20 = genome B (1–10). The -log_10_(p) for the association between SNP loci and pod constriction are plotted along the y-axis. The red line indicates the cut-off for significance at *p* ≤ 10^–4.76^

Physical localization of VPC and PCI in the peanut diploid genomes was done by TASSEL. Prior to trait-mapping, the adjusted association of 2882 SNPs with the pod constriction traits (VPC and PCI) was examined using quantile-quantile plots (Additional file 1: Figure S2). The quantile-quantile plots of both VPC and PCI were found to deviate from a uniform distribution, indicating that significant associations exist between SNPs and both traits. These associations were further confirmed by the physical mapping of VPC and PCI (Fig. 3). As shown in the plots, most of the SNPs associated with VPC and PCI were found in the same region at the end of chromosome B7. With the threshold of -log_10_(p) ≥ 4.76 (red line in Fig. 3a, b), 21 and 15 SNPs were significantly linked with VPC and PCI, respectively. One significant exception was a VPC-associated SNP found on chromosome 8 (Fig. 3a). The -log_10_(p) of the significant SNPs for VPC ranged from 15.20 to 4.85 and the total phenotypic variation explained by SNP markers (*R*^2^) ranged from 0.32 to 0.11 (Additional file 1: Table S1). PCI -log_10_(p) values ranged from 13.79 to 4.78 and total phenotypic variation explained by SNP markers (*R*^2^) ranged from 0.30 to 0.11.

**Fig. 4:**
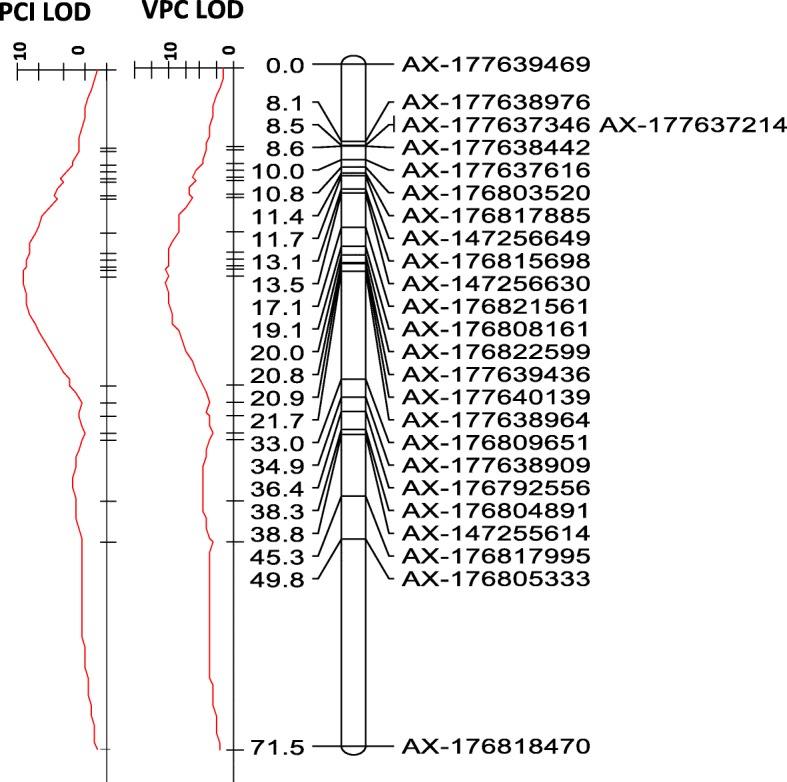
Genetic mapping of PCI and VPC on linkage group 15 and comparison to the physical position. Distances between markers are indicated in cM, to the left of the linkage group. LOD scores for the links between the SNPs and the traits are presented in the red graph on the left

Genetic mapping of VPC and PCI was performed to evaluate the original locations of the SNPs by their recombination rate in the population and particularly to validate the order of the SNPs within chromosome B7. A total of 19 linkage groups were generated with a 1570.16 cM map (Additional file 2: Table S2). Notably, the linkage groups of the genetic map did not match the original SNPs locations; many SNP markers originally considered to be A- or B-derived [14, 15] were mapped to the same linkage group (Additional file 2: Table S2). VPC and PCI were mapped to a single QTL at linkage group 15 at closely linked SNPs between markers B07_120,287,958 and B07_120,699,791, with best LOD scores of 10.17 and 9.95, respectively (Fig. 4). In contrast to most of the linkage groups, the order of the SNPs within this linkage group was almost completely in accordance with their expected physical order on chromosome B7, with only minor discrepancies and the inclusion of a number of SNPs from chromosomes A7 and A8. The SNP marker AX176792556, which was originally located at A8_1053912, and was found to be significantly linked to VPC (Fig. 3a), was found within linkage group 15.

## Discussion

In the current study, the inheritance pattern of the pod constriction trait was investigated in Virginia-type peanut. Pod constriction is a very important trait in the in-shell market. Occasionally breeders choose not to commercialize highly performing breeding lines for that market only because of excessive pod constriction, which causes pods to break during harvest, handling and shipping. The identity of genes controlling the pod constriction trait will allow breeders to apply marker-assisted breeding approaches to develop new genotypes with slighter pod constriction, alleviating the commercial industry of potential issues with splitting and aesthetics presented by some cultivars like cv. Hanoch. We used closely related Virginia-type peanut germplasm to study this trait against a homogenous genetic background. Therefore, in contrast to earlier studies based on crosses of relatively distantly related parents with extreme phenotypes (i.e., very slight vs. very deep pod constriction), the current study focused on less extreme phenotypes (i.e., slight vs. deep pod constriction).

Indeed, the result of 1:1 segregation of the trait indicates that against this genetic background, pod constriction may be controlled by a major gene, regardless of whether it was phenotyped visually or quantitatively. This is in contrast to the findings of most other pod constriction inheritance studies, which have indicated that there are several genes that control this trait in peanut [8–10, 12]. The explanation for this contrast may be the genetic material of the current study that is differing only in one locus while the other possible loci were not detected as an effect of selection (since two closely related cultivars were crossed). The slight pod constriction phenotype was dominant over the deep pod constriction phenotype, as shown by the segregation of this trait in the early F_1_/F_2_ generations. Interestingly, while the difference between the PCI values of the two parental lines was not particularly great, many RILs had significantly lower PCI values that indicate very deep pod constriction. This unexpected deep pod constriction phenotype suggests a possible environmental effect on the trait, the epistatic contribution of additional minor genes or a combination of those two factors.

A common characteristic of all of the above-mentioned studies is that they were based on visual, as opposed to quantitative evaluations of pod constriction. Here, pod constriction was evaluated both visually, by inspecting and grading the entire sample, and quantitatively, by measuring the ratio between the waist and shoulders (PCI) of randomly selected pods. It was found that, in general, no large differences exist between the two methods (R^2^ = 0.84) and data sets collected using both methods suggested that pod constriction is controlled by a major gene and, in both analyses, that putative gene was mapped to the same genomic location. Since these ratings were similar, the visual rating for pod constriction would be more efficient while evaluating many breeding plots. Yet, our results (as shown in Fig. 2) indicate that quantitative measurements of PCI may be more accurate than visual assessments. Therefore, the quantitative measurement should be the preferred method for tracking pod constriction in different backgrounds as well as for the identification of additional loci controlling the pod constriction trait in peanut.

SNP-array-based technology was used to map the VPC trait in the RIL population and identified a single locus for the VPC trait on chromosome B7. A large discrepancy was found between the genetic map and the original SNP localization on the A and B sub-genomes, since many A- and B-originating SNPs were assigned to the same linkage group. In some cases it was difficult to pair the linkage group to its corresponding physical chromosome. This observation, together with those of other studies that have used this SNP array for trait-mapping, indicates that it is important to re-address the positions of the SNPs by genetic mapping. Fortunately, linkage group 15 was constructed mostly from SNPs from B7, enabling us to locate PC and PCI in a small linkage group between markers B07_120,287,958 and B07_120,699,791. Some discrepancy was also found between the SNP original position and the genetic map of the traits within linkage group 15, indicating that there may be some microsyntenic differences in this region between the diploid and the tetraploid species.

An interesting outcome of the mapping analysis was that VPC and PCI were mapped to a single locus, but with relatively low -log_10_(p) and a relatively low LOD score. The best-linked SNP explained 32% of the total variation and a LOD score of ~ 10 (for VPC), which could be considered low for a major gene effect. This may be due to insufficient resolution of the SNP markers in this region. For example, although the genetic map showed that the pod constriction locus is located between B07_120,287,958 and B07_120,699,791, a large gap in SNP density was noted just below B07_120,287,958, with no downstream markers until B07_114704092 (Fig. 4). It is also possible that more minor loci also affect pod constriction in this background, which was not diagnosed significant in our relative strict Bonferroni cutoff of *p*-value ≤10^–4.76^ (Fig. 3).

According to the assumption that the VPC locus is indeed located within this ~ 400-kb region, an additional analysis of candidate genes was performed. Fifteen gene models were collected from this segment (Additional file 1: Table S3) using the peanut genome database [17]. Most of the genes did not match any of the gene products that have been associated with pod shape or fruit shape in the literature. However, one gene, a MADS-box transcription factor 1 located in the middle of this linkage group, may play a role in controlling VPC, since we speculate that the new pod shape should be controlled by multiple gene expression factors. Interestingly, this gene was previously found to be expressed solely in the pistil [18]. We hypothesize that the controller of pod constriction would be expressed much earlier in pod development, possibly even during the development of the female gametophyte or the fertilization process. Moreover, BLASTing this gene to the NR_PubMed database yielded two best-hits that are both Agamous-like MADS-box proteins, AGL104 and AGL11, with 70% and 67% identity, respectively. AGL11 is a transcription factor that is required for the maternal control of the formation of endothelium, which is the tissue that surrounds the seeds during their development [19]. This gene should be studied further, to examine its hypothetical role as a controller of pod constriction.

## Conclusion

The trait distribution and mapping that are presented in the current study, together with data from F_1_ and F_2_ generations, indicate that in this background the pod constriction is controlled by a major recessive gene. The identity of loci controlling the pod constriction trait will allow breeders to apply marker-assisted breeding approaches to shift allelic frequencies towards a slighter pod constriction and will facilitate future effort for map-based gene cloning.

## Methods

### Plant material and mapping populations

A recombinant inbred line population (F_7_RIL) that was derived from the cross of cv. Hanoch and cv. Harari, two closely related Israeli Virginia-type cultivars, was developed [20] and used for the study. ‘Hanoch’ has been the leading Israeli in-shell peanut cultivar for over two decades. It has long, smooth pods and a unique flavor, which made it very popular in the market. A major drawback of this cultivar is its relatively deeply constricted pod, which contributes to its “long shape” appearance, but also makes it vulnerable to pod-splitting. ‘Harari’, another Virginia-type cultivar, is characterized by smaller pods with moderate-to-slight pod constriction.

RILs were planted in April 2016 on broad bed furrow (two rows, 75 × 40 cm) in Nirim Magar, a location in southern Israel that is characterized by fine-sandy loam soil. Each RIL was replicated three times (randomized complete block design), with 16 plants for each RIL in each block. Plots were maintained under full-irrigation conditions and all recommended agronomic practices were carried out as described [20]. ‘Hanoch’ and ‘Harari’ plants were also grown as controls. Pods from each RIL and the parental cultivars were harvested manually by randomly collecting 300 sound and fully mature pods from the plants of each plot. In addition to the RIL population, F_2_ generation that was derived from the same cross was validated for PC in field trial based on individual plants. All field procedures for this population were previously described [16].

### Phenotyping of the pod constriction trait

For each RIL, the level of constriction was determined in two ways. First, the phenotype was determined visually by inspecting the entire sample and grading the pod constriction of each of the F_7_RILs as “deep” or “slight” (groups 1 or 3 in Additional file 1: Figure S1). Second, a random 30 pods were sampled from each RIL and a Vernier caliper was used to measure the breadth of the top and bottom shoulders of each pod, as well as the width of each pod’s constricted (waist) area. The pod constriction index (PCI) was calculated from mean values using the following formula:

$$ PCI=\frac{Waist\ width\ (mm)}{\left( Top+ bottom\ shoulder\ width\ (mm)\right)/2} $$

The measured and visual assessments of the pod constriction of the 195 members of the F_7_RIL population, as well as the frequency distribution of the pod constriction ratios, were analyzed using JMP® version 10 (SAS Institute; Cary, NC, USA). A chi-square (χ^2^) test was used to study the segregation pattern of pod constriction traits.

### Genotyping and SNP filtering

Young leaflets from each F_7_RIL were collected and genomic DNA was extracted using DNeasy® Plant Mini Kit (Qiagen; Hilden, Germany). Precise DNA quantification was carried out with Qubit (Invitrogen; CA, USA) and the samples were diluted to 30 ng/μL according to the Affymetrix guidelines. The second edition of the Axiom_*Arachis* array (Axiom_*Arachis*2) designed by Ozias-Akins lab, which is an improved version of the previously reported 58 k Axiom_Arachis SNP-array [14, 15], was used with 47,837 SNPs to genotype the 197 individuals (two parents and 195 F_7_ RILs). Genotyping data were analyzed by the Axiom analysis suite. The software output of the genotyping data in five categories based on the quality of signal clustering i.e. polyhighresolution (three clusters with good separation, major allele, minor allele and heterozygous state, minor allele at least detected two times), nominorhom (two clusters, minor allele is not present in homozygous state), monohighresolution (marker monomorphic, minor allele with zero or one copy), CallRateBelow Threshold (SNP call rate is below 90%, but other cluster properties are above) and Off-Target Variant categories (unexpected new alleles). Genotyping data was only extracted from polyhighresolution and nominorhomo categories since monohighresoultion class did not contain any polymorphic markers and most of the markers in the CallRateBelow Threshold and Off-Target Variant categories were ambiguous with signal clustering. The polymorphic homozygous SNPs (AA and BB) and polymorphic heterozygous SNPs (AA or BB and AB) were retained with 65–35% call-rate frequencies among the RILs. Two thousand eight hundred and eighty-two polymorphic SNPs were retained for further analyses.

### Physical localization of pod constriction trait in the peanut diploid genomes

The pod constriction trait was mapped based on the Axiom_*Arachis*2 SNP markers. TASSEL 5 [21] was used to test the association of the pod constriction trait with each of the 2882 SNPs across the peanut diploid genomes [22]. First, a general linear model was run and all of the probabilities generated in the association runs were transformed by -log_10_(p). Scores for each chromosome were then inspected in Manhattan plots to determine whether the SNPs reached the significance threshold for visual and quantitative measurements of pod constriction. The critical *p*-values for assessing the significance thresholds for the SNPs were corrected for multiple comparisons based on the Bonferroni method [23]; which gave the adjusted p-value of -log10 (α / *n*); α = significance level and *n* = the number of markers (2882 SNPs). The Bonferroni adjusted cut-off for accepting thresholds was set to -log_10_(p) ≥ 4.76, which corresponds to an experiment-wise error rate of 0.05.

### Genetic map construction and QTL analysis

Genetic maps were constructed using Join-Map 4.1 software [24]. From the error-free datasheet, a population node was created for 2849 loci (33 duplicated SNPs were excluded from the total 2882 SNPs) and 195 “individuals” (RILs). The multipoint maximum-likelihood mapping algorithm [25] was used to calculate locus genotype frequency, remaining only SNPs with chi-square p-value ≤0.05 (1 degree of freedom). Resulting the chi-square test, 723 loci were excluded for exceeding the threshold. Out of 4,056,976 pairs, 6112 were excluded due to loci-similarity. Groupings were established according to an independent LOD that started at 2.0 and ended at 10. Map distances were estimated using Haldane’s (1919) mapping function. QTL mapping analyses of the 195 F_7_RILs were performed using MapQTL 6 [26] for the visual (VPC) and quantitative (PCI) pod constriction traits. Loci were detected by interval mapping using significance threshold levels derived from permutation tests. A LOD score of 2.5 with 1000 permutations was used to confirm the presence of a putative QTL.

### Statistical analysis

The analysis of variance was carried out according to the standard procedure described by Panse and Sukhatme [27]. The statistical model for the randomized block design used for the analysis of variance was:

$$ {Y}_{ij}=\mu +{r}_i+{\mathrm{g}}_j+{\delta}_{ij} $$

In that model, *Y*_*ij*_ is the response of the *j*^th^ RIL in the *i*^th^ block, *μ* is the general mean, *r*_*i*_ is the effect of the *i*^th^ block, *g*_*j*_ is the effect of the *j*^*t*h^ RIL and *δ*_*ij*_ is the random residual error associated with the *j*^th^ genotype in the *i*^th^ replication.

The genetic variability, heritability and genetic advance parameters were calculated in a similar method as previously described [20]. Briefly, environmental, genotypic and phenotypic coefficients of variation (ECV, GCV and PCV) for existing in traits were estimated using the formula:

$$ ECV\left(\%\right)=\frac{\sqrt{{\widehat{\upsigma}}_{\mathrm{e}}^2}}{\overline{\mathrm{X}}}\times 100,\kern0.5em GCV\left(\%\right)=\frac{\sqrt{{\widehat{\upsigma}}_g^2}}{\overline{\mathrm{X}}}\times 100,\kern0.5em PCV\left(\%\right)=\frac{\sqrt{{\widehat{\upsigma}}_p^2}}{\overline{\mathrm{X}}}\times 100 $$

wherein, ${\sigma}_p^2$, ${\sigma}_g^2$, and ${\sigma}_e^2$ are the phenotypic, genotypic and environmental variance components [20], respectively, and X is the general mean.

Heritability *(h*^*2*^*)* in broad sense was calculated according to the formula:

$$ {h}^2=\frac{{\widehat{\sigma}}_g^2}{{\widehat{\sigma}}_p^2}\times 100 $$

The expected genetic advance (GA) was measured using the formula:

$$ GA=\frac{{\mathrm{V}}_{\mathrm{g}}}{{\mathrm{V}}_{\mathrm{p}}}\times \sqrt{{\mathrm{V}}_{\mathrm{p}}}\times \mathrm{k},=\frac{{\mathrm{V}}_{\mathrm{g}}}{\sqrt{{\mathrm{V}}_{\mathrm{p}}}}\times \mathrm{k} $$

In that formula, *V*_*g*_ is the genotypic variance, *V*_*p*_ is the phenotypic variance and *k* is the selection differential (constant; i.e., 2.06 at 5% selection intensity).

## Additional files

Additional file 1: **Figure S1.** Visual pod constriction descriptors for peanut. Cv. Hanoch and Harari belong to Deep (1) and Slight-Moderate (3) groups, respectively. **Figure S2.** Quantile–quantile plot of visually assessed pod constriction (VPC) and mean pod constriction index (PCI) values for 195 F_7_ RILs. **Table S1**. Significant SNP markers associated with visual (VPC) and quantitative (PCI) assessments of the pod constriction trait. **Table S3.** List of gene models within the B07:120287958…120,699,791 genomic segment. (PDF 398 kb)

Additional file 2: **Table S2.** Peanut linkage groups constructed based on 195 F_7_ RILs. (XLSX 75 kb)

## Abbreviations

PCI: Pod constriction index
QTL: Quantitative trait locus
RIL: Recombinant inbred line
VPC: Visual pod constriction
