# Supplementary material for: Genetic insight and mapping of the pod constriction trait in Virginia-type peanut
Source: BMC Genet. 2018 Oct 19;19:93. doi: 10.1186/s12863-018-0674-z (PMC6195699; doi:10.1186/s12863-018-0674-z)
Supplement: Supplementary file 1 — Figure S1. Visual pod constriction descriptors for peanut. Cv. Hanoch and Harari belong to Deep (1) and Slight-Moderate (3) groups, respectively. Figure S2. Quantile–quantile plot of visually assessed pod constriction (VPC) and mean pod constriction index (PCI) values for 195 F7 RILs. Table S1. Significant SNP markers associated with visual (VPC) and quantitative (PCI) assessments of the pod constriction trait. Table S3. List of gene models within the B07:120287958…120,699,791 genomic segment. (PDF 398 kb) [file 12863_2018_674_MOESM1_ESM.pdf]

Supplementary material

Figure S1

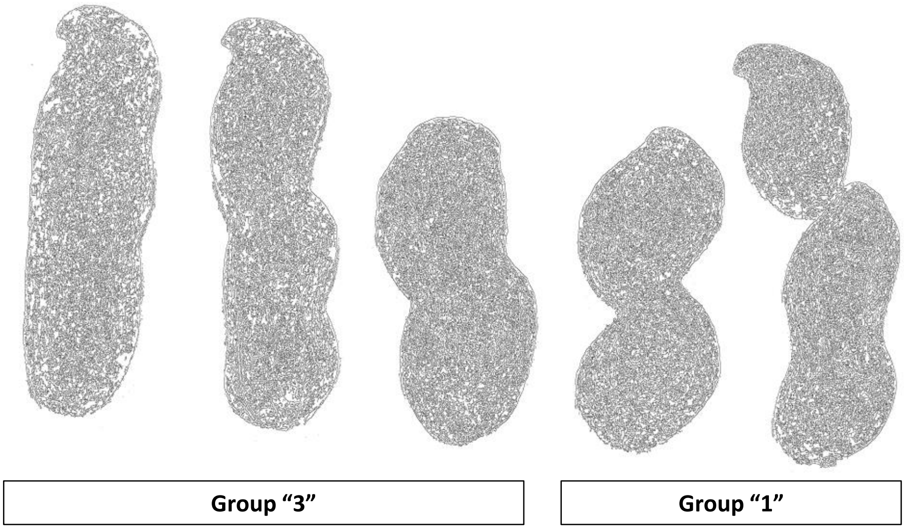

Figure S2

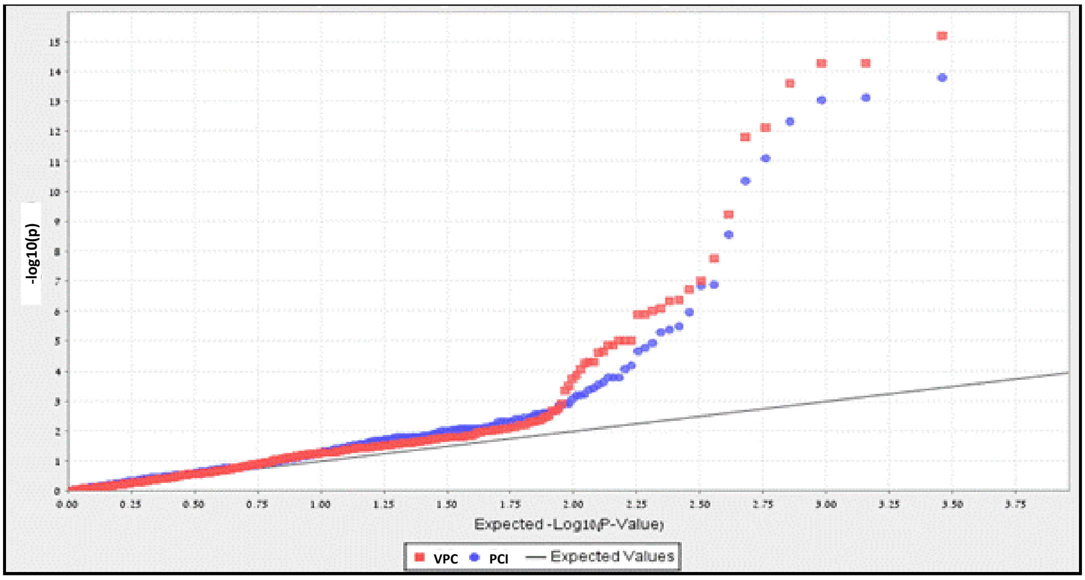

Table S1. Significant SNP markers associated with visual (VPC) and quantitative (PCI) assessments of the pod-constriction trait

Trait	Probe ID	Chromosome	Position(bp)	*p*-value	$-\log_{10}(p)$	Marker R^2
VPC	AX177638964	17	120287958	6.2E-16	15.20	0.32
VPC	AX177640139	17	120268599	5.1E-15	14.29	0.29
VPC	AX177639436	17	120420782	5.3E-15	14.28	0.29
VPC	AX176822599	17	120699791	2.4E-14	13.61	0.28
VPC	AX177639347	17	120550537	7.6E-13	12.12	0.24
VPC	AX176808161	17	120838334	1.6E-12	11.81	0.26
VPC	AX176821561	17	121098593	5.9E-10	9.23	0.21
VPC	AX176815698	17	122055610	1.8E-08	7.76	0.18
VPC	AX176822637	17	122247802	9.6E-08	7.02	0.15
VPC	AX147256630	17	121954137	1.9E-07	6.72	0.15
VPC	AX147256649	17	122177705	4.3E-07	6.36	0.14
VPC	AX176817885	17	122260525	4.6E-07	6.34	0.14
VPC	AX177637980	17	122128386	8.0E-07	6.10	0.13
VPC	AX176806530	17	30845972	9.4E-07	6.03	0.12
VPC	AX176809651	17	114704092	1.3E-06	5.90	0.13
VPC	AX176817995	17	16412296	1.3E-06	5.89	0.13
VPC	AX176792556	8	1053912	9.7E-06	5.01	0.11
VPC	AX147256000	17	102224525	9.7E-06	5.01	0.11
VPC	AX176819626	17	106063047	9.7E-06	5.01	0.11
VPC	AX176803520	17	122476073	1.4E-05	4.86	0.11
VPC	AX177638909	17	111731434	1.4E-05	4.85	0.11
PCI	AX177638964	17	120287958	1.6E-14	13.79	0.30
PCI	AX177640139	17	120268599	7.5E-14	13.12	0.27
PCI	AX177639436	17	120420782	8.8E-14	13.05	0.27
PCI	AX176822599	17	120699791	4.6E-13	12.34	0.26
PCI	AX177639347	17	120550537	8.0E-12	11.10	0.22
PCI	AX176808161	17	120838334	4.4E-11	10.36	0.24
PCI	AX176821561	17	121098593	2.7E-09	8.57	0.20
PCI	AX176815698	17	122055610	1.3E-07	6.87	0.16
PCI	AX176822637	17	122247802	1.4E-07	6.86	0.15
PCI	AX147256630	17	121954137	1.1E-06	5.95	0.13
PCI	AX147256649	17	122177705	3.3E-06	5.49	0.12
PCI	AX177637980	17	122128386	4.1E-06	5.39	0.12
PCI	AX176817885	17	122260525	5.0E-06	5.30	0.12
PCI	AX176806530	17	30845972	1.2E-05	4.93	0.10
PCI	AX176809651	17	114704092	1.7E-05	4.78	0.11

Table S3. List of gene models within the B07:120287958…120699791 genomic segment.

Genome location	Gene model
Araip.B07:120285940..120287411	Predicted methyltransferase
Araip.B07:120364965..120365903	Uncharacterized protein LOC102665249 isoform X4
Araip.B07:120373162..120374726	Tetratricopeptide repeat (TPR)-like superfamily protein
Araip.B07:120394118..120406510	Myosin heavy-chain-related protein
Araip.B07:120408286..120409428	Galactose oxidase/kelch repeat superfamily protein;
Araip.B07:120465390..120466687	Heavy metal transport/detoxification superfamily
Araip.B07:120468417..120472189	MADS-box transcription factor 1
Araip.B07:120475403..120478130	Poly [ADP-ribose] polymerase 3
Araip.B07:120512267..120514427	Serine/threonine-protein phosphatase 7 long-form homolog
Araip.B07:120517039..120518963	Uncharacterized protein LOC100797259 isoform X4
Araip.B07:120537272..120546148	Uncharacterized protein LOC102661750 isoform X2 [*Glycine max*]
Araip.B07:120552289..120559291	Sulfate transporter 1; 3;
Araip.B07:120590131..120594404	Sulfate transporter 1; 3
Araip.B07:120595882..120599920	tRNA modification GTPase, putative
Araip.B07:120600484..120613531	Acetyl-CoA carboxylase 1
